# Green nail syndrome caused by *Citrobacter braakii*

**DOI:** 10.1002/ccr3.4203

**Published:** 2021-05-24

**Authors:** Francesk Mulita, Levan Tchabashvili, Elias Liolis, Konstantinos Tasios, Fotios Iliopoulos, Charalampos Kaplanis, Nikolaos Parchas, Kerasia‐Maria Plachouri

**Affiliations:** ^1^ Department of General Surgery General University Hospital of Patras Patras Greece; ^2^ Department of Internal Medicine General University Hospital of Patras Patras Greece; ^3^ Department of Orthopedics General University Hospital of Patras Patras Greece; ^4^ Department of Dermatology General University Hospital of Patras Patras Greece

**Keywords:** *Citrobacter braakii*, green nail syndrome, onychectomy, onycholysis

## Abstract

A 34‐year‐old woman presented due to progressive painful swelling around the nail of the right index finger. Onychectomy and drainage of the abscess of the affected finger were performed as the inflammation was progressive despite the previous antibiotic therapy. The microbiological culture revealed a ciprofloxacin‐susceptible Citrobacter braakii.

## Graphical abstract

Green nail syndrome (GNS) is a rare condition, usually caused by *Pseudomonas aeruginosa*. A case of GNS caused by *Citrobacter braakii* has not been reported in the literature so far.

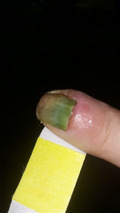

## 1 CASE DESCRIPTION

A 34‐year‐old woman presented in our emergency department due to progressive painful swelling around the nail of the right index finger, that had first appeared 30 days prior to the referral. The symptoms occurred immediately after a finger injury. Approximately 2 weeks after the trauma, the patient noticed a greenish discoloration of the nail plate of the affected finger. An empirical antibiotic therapy with amoxicillin/clavulanic acid had been unsuccessful.

**FIGURE 1:**
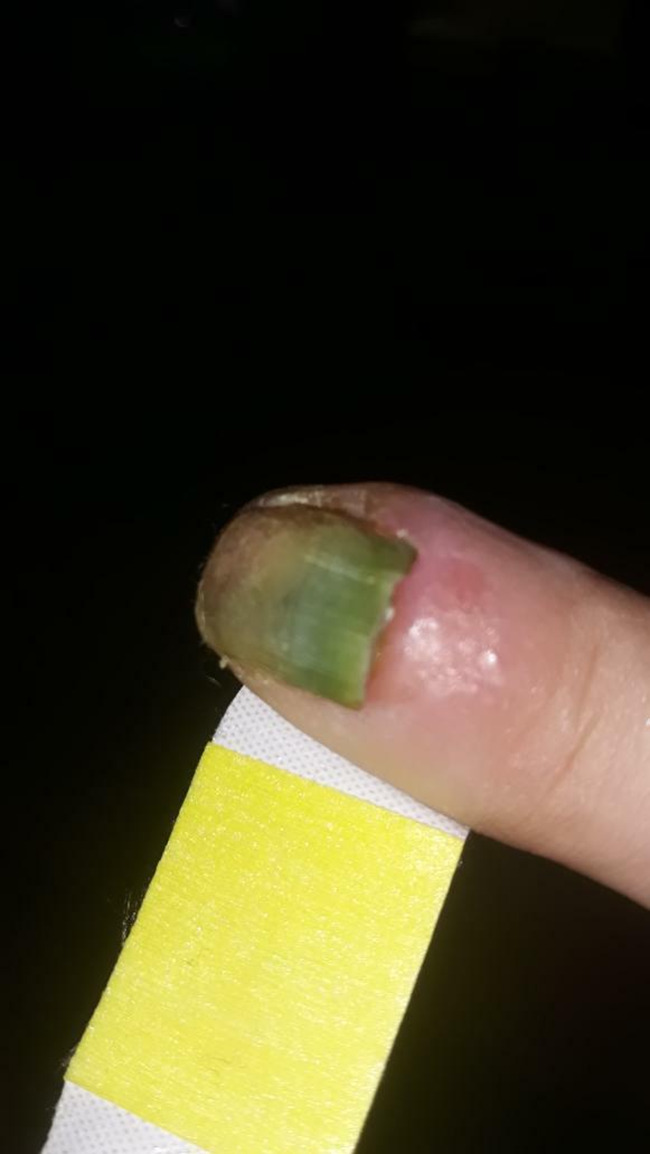
Green nail syndrome in a 34‐y‐old young woman: A distal onycholysis and a greenish discoloration of the entire nail plate of the right index finger, as well as a purulent proximal nail fold

On examination, the patient's vital signs were unremarkable. A distal onycholysis and a greenish discoloration of the entire nail plate of the right index finger, as well as a purulent proximal nail fold were seen (Figure 1).

Onychectomy and drainage of the abscess of the affected finger were performed as the inflammation was progressive despite the previous antibiotic therapy. The microbiological culture revealed a ciprofloxacin‐susceptible *Citrobacter braakii* infection, and a postoperative antibiotic therapy with ciprofloxacin was, therefore, prescribed. In the 5‐day follow‐up, the patient was symptom‐free and the inflammatory markers (WBC, CRP) were within the normal range.

Green nail syndrome (GNS) is a rare condition, usually caused by *Pseudomonas aeruginosa*.^1^ However, other pathogens, such as the rare gram‐negative bacillus *C* *braakii,* can also be involved in the pathogenesis of this condition.^2^

## CONFLICT OF INTEREST

None declared.

## AUTHOR CONTRIBUTIONS

FM, LT, EL, KT, FI, CK, NP, and K‐MP: contributed to the clinical data collection and prepared the case report. FM and K‐MP: contributed to the design of the case report presentation and performed the final revision of the manuscript.

## PATIENT CONSENT FOR PUBLICATION

A written informed consent was obtained from the patient for publication of this case report.

## Data Availability

Data are available on request from the authors.
